# Genome-scale case-control analysis of CD4+ T-cell DNA methylation in juvenile idiopathic arthritis reveals potential targets involved in disease

**DOI:** 10.1186/1868-7083-4-20

**Published:** 2012-11-13

**Authors:** Justine A Ellis, Jane E Munro, Raul A Chavez, Lavinia Gordon, Jihoon E Joo, Jonathan D Akikusa, Roger C Allen, Anne-Louise Ponsonby, Jeffrey M Craig, Richard Saffery

**Affiliations:** 1 Genes, Environment & Complex Disease, Murdoch Childrens Research Institute, 50 Flemington Rd, Parkville, Vic, Australia; 2 Rheumatology, Department of General Medicine, Royal Children’s Hospital, Parkville, Vic, Australia; 3 Arthritis & Rheumatology, Murdoch Childrens Research Institute, Parkville, Vic, Australia; 4 Bioinformatics Unit, Murdoch Childrens Research Institute, Parkville, Vic, Australia; 5 Early Life Epigenetics, Murdoch Childrens Research Institute, Parkville, Vic, Australia; 6 Cancer, Disease and Developmental Epigenetics, Murdoch Childrens Research Institute, Parkville, Vic, Australia; 7 Environmental & Genetic Epidemiology Research, Murdoch Childrens Research Institute, Parkville, Vic, Australia; 8 Department of Physiology, The University of Melbourne, Melbourne, Vic, Australia

**Keywords:** Epigenetics, Juvenile idiopathic arthritis, DNA methylation, Autoimmunity, Methylome, Methotrexate

## Abstract

**Background:**

Juvenile Idiopathic Arthritis (JIA) is a complex autoimmune rheumatic disease of largely unknown cause. Evidence is growing that epigenetic variation, particularly DNA methylation, is associated with autoimmune disease. However, nothing is currently known about the potential role of aberrant DNA methylation in JIA. As a first step to addressing this knowledge gap, we have profiled DNA methylation in purified CD4+ T cells from JIA subjects and controls. Genomic DNA was isolated from peripheral blood CD4+ T cells from 14 oligoarticular and polyarticular JIA cases with active disease, and healthy age- and sex-matched controls. Genome-scale methylation analysis was carried out using the Illumina Infinium HumanMethylation27 BeadChip. Methylation data at >25,000 CpGs was compared in a case-control study design.

**Results:**

Methylation levels were significantly different (FDR adjusted p<0.1) at 145 loci. Removal of four samples exposed to methotrexate had a striking impact on the outcome of the analysis, reducing the number of differentially methylated loci to 11. The methotrexate-naive analysis identified reduced methylation at the gene encoding the pro-inflammatory cytokine IL32, which was subsequently replicated using a second analysis platform and a second set of case-control pairs.

**Conclusions:**

Our data suggests that differential T cell DNA methylation may be a feature of JIA, and that reduced methylation at *IL32* is associated with this disease. Further work in larger prospective and longitudinal sample collections is required to confirm these findings, assess whether the identified differences are causal or consequential of disease, and further investigate the epigenetic modifying properties of therapeutic regimens.

## Background

Juvenile Idiopathic Arthritis (JIA) is the most common autoimmune rheumatic disease of childhood. The aetiology of JIA remains largely unknown, but as for other autoimmune diseases, including adult rheumatoid arthritis, the interplay between genes and environment is likely to be important [1]. A potential conduit for such interactions to alter disease risk is via epigenetic modification, defined as “the structural adaptation of chromosomal regions so as to register, signal or perpetuate altered activity states” [2]. For example, methylation of CpG dinucleotides at gene promoters is generally associated with a reduction in gene expression. Folate, through one-carbon metabolism, is an important primary methyl donor (reviewed in [3]), and maternal folate intake has been shown to alter epigenetic profile and resultant phenotypes of offspring in rodents [4]. In humans, studies of monozygotic twin pairs have demonstrated modification of epigenetic profile across the life course in response to environmental exposure (reviewed in [5]).

A growing body of evidence suggests that epigenetic modification plays an important role in specifying risk associated with autoimmune disease [6]. Unequivocal evidence links epigenetics to regulation of the immune response. For example the expression of cytokines such as interleukin 4 (IL4) and interferon gamma (IFNγ) that drive naive T cells to differentiate into T helper 1 (Th1) or Th2 lineages is epigenetically controlled [6,7]. Immune tolerance is also associated with epigenetic changes, for example increased DNA methylation of the gene encoding IL2, an important T cell growth factor, leads to reduced IL2 expression and decreased T cell proliferation [8]. Epigenetic involvement in a number of autoimmune diseases has been considered; one of the most well studied is systemic lupus erythematosus (SLE), a disease associated with global T cell and B cell hypomethylation [9] and changes to the methylation state of specific gene loci [10]. Aberrant DNA methylation has also been implicated in rheumatoid arthritis (RA), with reduced DNA methylation reported in peripheral blood mononuclear cells (PBMC) [11], T cells [12] and synovial cells [13], including at a specific CpG site at the gene encoding the pro-inflammatory cytokine IL6 [14]. Hypermethylation of the promoter region of the gene encoding death receptor 3 (*DR3*), a member of the apoptosis-inducing tumor necrosis factor (TNF) receptor superfamily, has also been reported in RA synovial cells [15].

Despite the mounting evidence for epigenetic disruption in autoimmune rheumatic diseases, no studies to date have considered its role in JIA. This may be important not only for understanding aetiology, but also because the modifiable nature of epigenetic marks makes them ideal therapeutic targets [16]. As a first attempt to address this knowledge gap, we have performed a genome-scale comparison of CD4+ T cell DNA methylation at over 27,000 CpG sites associated with over 14,000 genes in 14 children with active JIA (10 oligoarticular and 4 polyarticular) and 14 age- and sex-matched healthy control children.

## Results

### Differential methylation between cases and controls

**Figure 1:**
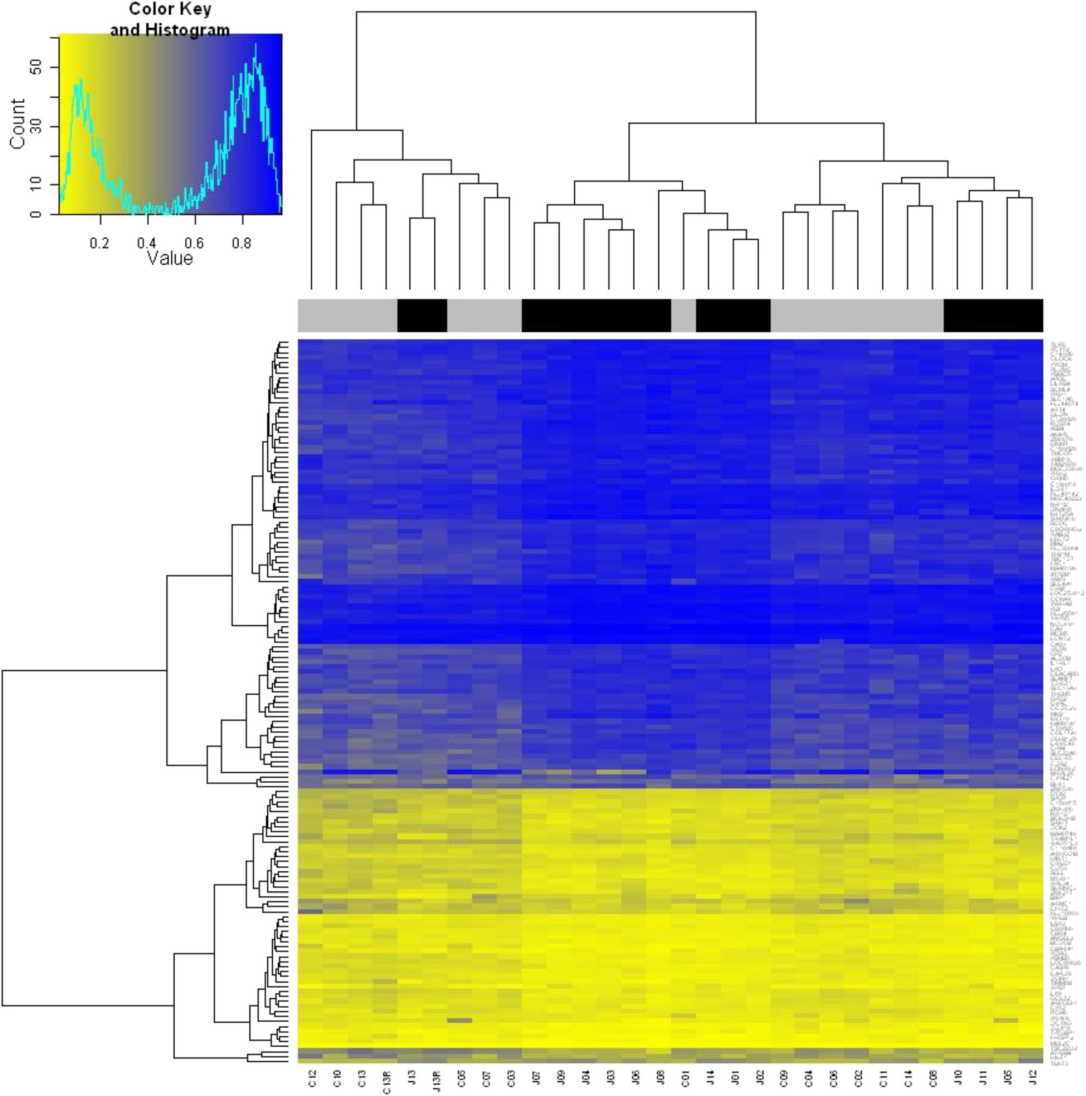
**Heatmap of significantly differentially methylated loci between JIA cases and controls.** Black = case, Grey = control. ‘Value’ = percent methylation.

Greater than 97% of HM27 probes passed QC measures with technical replicates demonstrating a high degree of reproducibility (Additional file 1: Figure S1). Following adjustment of p values for false discovery rate (FDR), probes associated with 145 genes were significantly different (adjusted p value < 0.1) between cases and controls (Table 1 a, Additional file 1: Table S1). A total of 91 genes were more highly methylated in cases, whilst 54 were more highly methylated in controls. Hierarchical clustering demonstrated that cases and controls did not cluster by phenotype (Figure 1), which might be expected for a complex disease such as JIA [1].

**Table 1:** **Genes associated with probes identified by Infinium HumanMethylation27K BeadChip array as significantly differentially methylated (a) between all cases and controls (20 most significant loci shown – for a full list refer to Additional file**1**: Table S2), and (b) between MTX-naïve cases and controls**

	**Gene**	**p value**	Adjusted p value*	**B**	**Case relative to control**	**Median controls**	**Median cases**	**Difference**
*(a)*	*ALDOB*	6.64E-05	0.069	1.727	Higher	0.715	0.791	0.075
	*C8ORFK32*	4.09E-05	0.067	2.146	Higher	0.754	0.794	0.040
	*DGKH*	6.38E-05	0.069	1.762	Lower	0.114	0.089	0.025
	*FLJ20581*	2.65E-05	0.067	2.519	Higher	0.877	0.924	0.047
	*FLJ36004*	4.65E-05	0.067	2.036	Higher	0.738	0.823	0.085
	*GCET2*	5.88E-06	0.067	3.803	Lower	0.152	0.107	0.044
	*GRM5*	3.36E-05	0.067	2.317	Higher	0.78	0.846	0.066
	*GYPE*	1.72E-05	0.067	2.893	Higher	0.688	0.781	0.093
	*LILRB4*	3.39E-05	0.067	2.309	Higher	0.823	0.866	0.043
	*LOC90826*	1.22E-05	0.067	3.185	Lower	0.142	0.091	0.051
	*MEF2C*	3.57E-05	0.067	2.263	Lower	0.075	0.056	0.019
	*MRPL28*	4.03E-05	0.067	2.159	Lower	0.875	0.685	0.190
	*NR1I2*	3.38E-05	0.067	2.311	Higher	0.842	0.89	0.047
	*PRH1*	2.84E-05	0.067	2.461	Higher	0.813	0.875	0.062
	*RAD51L3*	4.58E-05	0.067	2.049	Lower	0.277	0.202	0.075
	*Rgr*	4.12E-06	0.067	4.102	Higher	0.894	0.931	0.037
	*SCOC*	4.74E-05	0.067	2.019	Higher	0.765	0.825	0.060
	*SLC13A3*	2.35E-05	0.067	2.624	Higher	0.712	0.784	0.073
	*TIMM8B*	1.56E-05	0.067	2.972	Lower	0.124	0.079	0.045
	*TRAM1L1*	4.51E-05	0.067	2.063	Lower	0.224	0.142	0.083
*(b)*	*PRKRA*	7.51E-07	0.019	4.225	Higher	0.061	0.063	0.002
	*HOXB4*	3.46E-06	0.044	3.177	Lower	0.073	0.064	0.009
	*C6orf203*	1.02E-05	0.049	2.415	Lower	0.095	0.084	0.011
	*HCRTR1*	1.20E-05	0.049	2.300	Higher	0.659	0.716	0.057
	*C9orf43*	1.23E-05	0.049	2.281	Higher	0.043	0.046	0.003
	*LIN37*	1.31E-05	0.049	2.239	Higher	0.031	0.032	0.001
	*MEP1A*	1.63E-05	0.049	2.084	Higher	0.882	0.927	0.045
	*NDUFB10*	1.79E-05	0.049	2.018	Higher	0.026	0.027	0.001
	*RCAN2*	1.82E-05	0.049	2.003	Higher	0.879	0.906	0.027
	*IL32*	1.91E-05	0.049	1.969	Lower	0.335	0.298	0.037
	*SOCS2*	2.35E-05	0.054	1.821	Lower	0.098	0.076	0.022

* P value following adjustment for false discovery rate (FDR).

B = log-odds that the probe is differentially methylated.

### Gene ontology and pathway analyses

Gene ontology analyses of gene-associated differentially methylated probes in cases versus controls identified dicarboxylic acid transport as a significantly perturbed biological process (p = 0.0004), the mitochondria as a significant cellular component (p = 0.0006), and the L-aspartate transmembrane transporter activity as a significantly perturbed molecular function (p = 0.0006).

IPA software was used to identify networks amongst the differentially methylated loci. Twenty one genes fell into a network for which the top function was ‘immunological disease’, 16 genes fell into a network for which the top function was identified as ‘cellular growth and proliferation’, and 15 genes fell into a network in which the top functions included ‘antigen presentation’ and ‘cell-to-cell signalling and interaction’. Genes falling into these classifications are shown in Table 2.

**Table 2:** Differentially methylated (DM) genes identified as falling within the top networks using IPA pathway analysis (a) between all cases and controls, and (b) between MTX-naïve cases and controls

	**Top network functions**	**# DM genes**	**Genes**
*(a)*	Immunological disease, cardiac hypertrophy, cardiovascular disease	21	*APOE, ATP2B4, CARD8, CASP8, CAV3, COL17A1, DOK2, ESR2, HRSP12, IL1RL1, ITGA6, MEF2C, NR1D1, NR1I2, PLUNC, PSMC5, RETN, SELL, SPA17, SPOP, TLR6*
	Cellular Growth and Proliferation, Gene Expression, Cellular Movement	16	*ANGEL2, ARMC1, C11orf49, CBARA1, FXC1, KIAA0196, KRT10, LCN12, MRPL28, MTRF1, NR1D1, PSMB5, PSMC5, RBM18, RSL24D1, TFB1M*
	Antigen Presentation, Cell-To-Cell Signaling and Interaction, Hematological System Development and Function	15	*AKAP6, ARFGEF1, BCKDHB, CDS2, CEACAM3, CPT2,CRB1, GCET2, GPD2, LY9, RAB32,RBP3, SLC1A6, TIMM8B, ZKSCAN3*
*(b)*	Cellular growth and proliferation, Haematological system development and function, Hematopoiesis	9	*HCRTR1, HOXB4, IL32, LIN37, MEP1A, NDUFB10, PRKRA, RCAN2, SOCS2*

### Consideration of JIA subtypes

In recognition of the potential genetic heterogeneity between the oligoarticular and polyarticular JIA subtypes [17], differential methylation between cases and controls was considered within the subtype groups. Only one locus, *SPATA16* (spermatogenesis associated 16), remained significant (P_adj_ = 0.056) when the 10 oligoarticular JIA cases (mean β = 0.84) were compared to their matched controls (mean β = 0.87). *SPATA16* has been associated with male infertility [18], but has no known relationship to arthritis or autoimmune disease.

Not surprisingly, given the limited sample size, no loci were significantly differentially methylated when the four polyarticular cases were compared to matched controls.

### An effect of methotrexate on DNA methylation profile in JIA?

In recognition of the potential impact of the disease modifying anti-rheumatic drug (DMARD) methotrexate (MTX, a folate metabolism inhibitor [19]), on DNA methylation, we subsequently removed four cases from the array data that had been exposed to MTX prior to the collection of T cells. Case-control comparisons were run again, resulting in a total of 11 significantly (P<0.1) differentially methylated probes following FDR adjustment, associated with 11 different genes (Table 1 b). These genes did not overlap with those identified by the original analysis. Hierarchical clustering based on these 11 loci did not clearly delineate cases from controls (Additional file 1: Figure S2).

**Figure 2:**
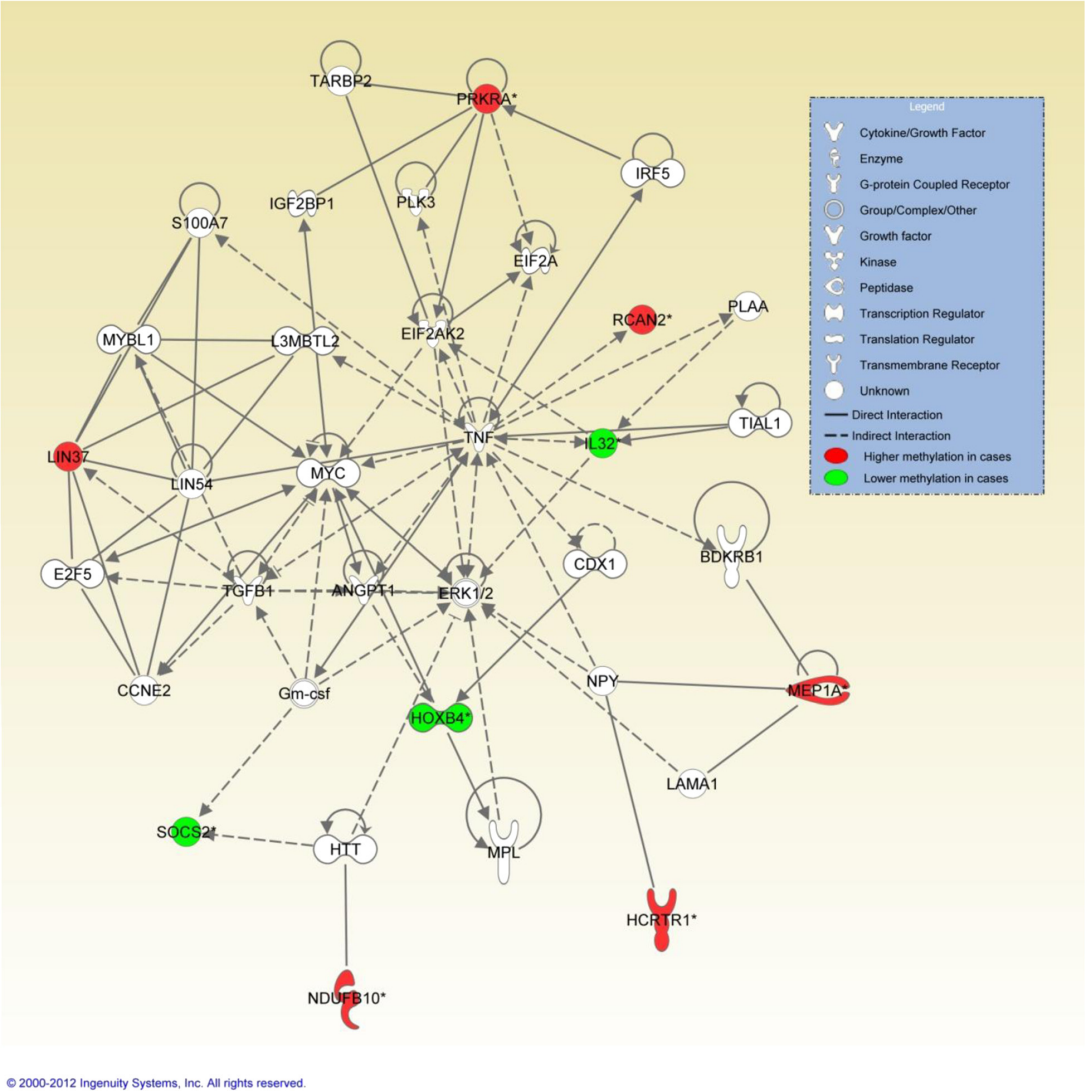
Ingenuity pathway analysis (IPA) top network that incorporates 9 genes identified as differentially methylated between methotrexate-naïve cases and controls by HM27 analysis.

The number of identified genes was insufficient for a meaningful GO analysis. IPA analysis of genes within this list identified ‘cellular growth and proliferation, haematological system development and function, hematopoiesis’ as the top network, involving 9 of the 10 characterised differentially methylated genes (Table 2, Figure 2). The top three biological functions were ‘connective tissue development and function’, ‘skeletal and muscular system development and function’, and ‘tissue development’. The top two identified canonical pathways were ‘IL9 signalling’, and ‘role of JAK2 in hormone-like cytokine signalling’.

### Validation of HM27 methylation differences and replication in an additional JIA sample

In order to confirm validity of the HM27 data, we measured gene-specific methylation of two differentially methylated genes, *MRPL28* and *IL32*, by Sequenom MassARRAY Epityper analysis. For validation, methylation was measured in the ‘discovery’ (array) case-control pair set (where sufficient DNA was available). Additionally, we attempted to replicate the observed differential methylation in a second set of 12 case-control pairs, the ‘replication’ pair set (Additional file 1: Table S2).

**Figure 3:**
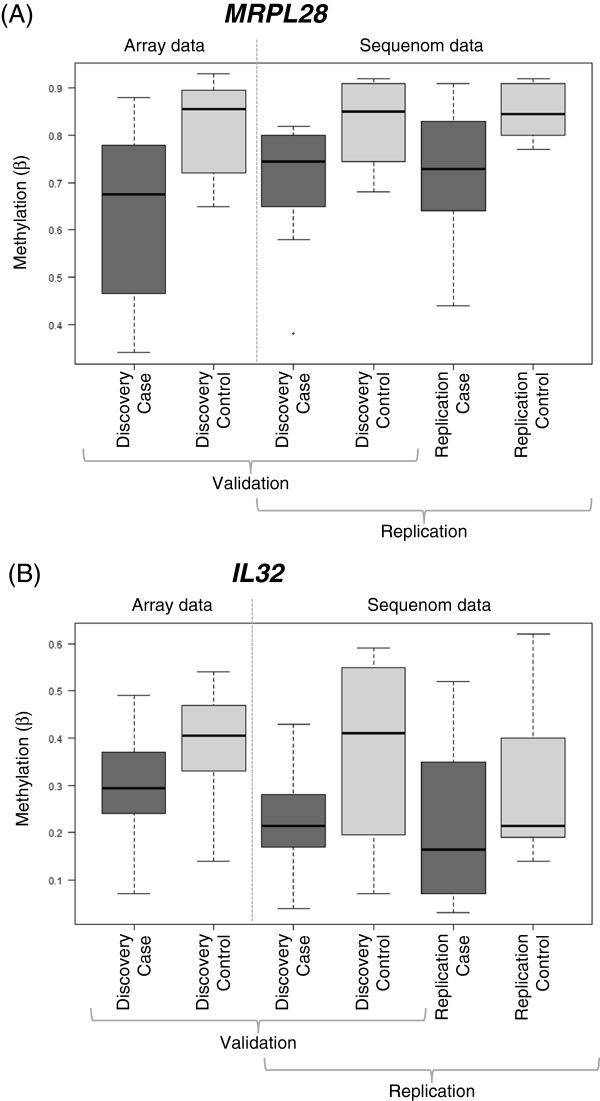
**Boxplots of validation and replication JIA case-control data for differentially methylated genes *MRPL28 *and *IL32*.** (**A**) HM27 *MRPL28* array data (probe cg12437481) for the discovery case-control pairs, and *MRPL28* sequenom data (assay unit CpG_14.15 – see Additional file 1: Figure S3) for the discovery and replication case-control pairs. (**B**) HM27 *IL32* array data (probe cg18350391) for the discovery case-control pairs, and *IL32* sequenom data (assay unit CpG_10 – see Additional File 1: Figure S4) for the discovery and replication case-control pairs. Comparing array and sequenom data for the discovery samples demonstrates validation of array data, and comparing sequenom data between the discovery and replication samples demonstrates replication of the direction of effect and magnitude of difference between the cases and controls at these loci

*MRPL28* was chosen for validation because of the large HM27 case-control β value differences observed (Figure 3A, Additional file 1: Table S3). We quantitatively measured methylation at 11 CpG dinucleotides within 8 assay units at *MRPL28* (Additional file 1: Figure S3), including the HM27 differentially-methylated probe (DMP) site (assay unit CpG_14.15). Across the entire dataset, β values for the HM27 DMP and assay unit CpG_14.15 were significantly correlated (r = 0.92, p <0.0001). Large case-control Δβ values were observed for several Sequenom CpG assay units, especially those lying in close proximity to the HM27 probe (Additional file 1: Table S3). Figure 3A presents boxplots of methylation values obtained from the array probe in the discovery case-control samples, and from Sequenom assay unit CpG_14.15 in the discovery and replication case-control samples. Conditional logistic regression demonstrated significant (p < 0.05) association of JIA with lower methylation levels at three of the measured CpG units, including CpG_14.15, such that a 1% increase in methylation equates to a 10% decrease in risk of being a JIA case (Odds Ratio (OR) = 0.90, 95% CI 0.81, 0.99; p = 0.026). For all Sequenom assay units, mean β values were lower in cases than controls, consistent with the direction of difference observed by HM27. Finally, this direction of difference was consistently observed when both the discovery and replication case-control pair groups were considered separately for all but one CpG unit measured (Additional file 1: Table S3), suggesting that such differences are genuinely associated with the JIA phenotype.

We also quantitatively measured methylation at 10 CpG sites within 9 assay units at the pro-inflammatory cytokine gene *IL32*. *IL32* was identified as differentially methylated between cases and controls following removal of MTX-exposed cases from analysis, and as a pro-inflammatory cytokine, was considered a physiologically plausible candidate gene. The HM27 DMP was directly captured by the Sequenom assay (CpG_10, see Additional file 1: Figure S4). Methylation was measured in both the discovery and replication pair sets following removal of MTX-exposed pairs 1, 4, 10, 13, and 19 (Additional file 1: Table S2). Across the entire dataset, β values for the HM27 differentially methylated probe and assay unit CpG_10 were significantly correlated (r = 0.70, p = 0.0008). Case-control Δβ values were largest for CpG_10 and neighbouring CpG_9; this was most striking in the discovery pair set, but of consistent direction in the replication pair set (Additional file 1: Table S4). Figure 3B presents boxplots of methylation values obtained from the array probe in the discovery case-control samples, and from Sequenom assay unit CpG_9 in the discovery and replication case-control samples. Conditional logistic regression of the combined dataset demonstrated nominally significant association of JIA with lower methylation at CpG_9, such that a 1% increase in methylation equates to a 6% decrease in risk of being a JIA case (OR = 0.94, 95% CI: 0.87, 1.00; p = 0.064). Significant associations were not detected when the discovery or replication datasets were considered separately, although the direction of effect was the same (discovery sample OR = 0.92, 95% CI: 0.83, 1.03, p = 0.13; replication sample OR = 0.95, 95% CI: 0.87, 1.04, p = 0.26), suggestive of replication and genuine association.

## Discussion

We present the first report of genome-scale analysis of DNA methylation profile in JIA. We have identified 145 differentially methylated loci of which 91 are more highly methylated in cases. Significant case-control differences are generally modest (difference in median β ranging from 0.006 – 0.19), however, this is consistent with a growing number of other equivalent studies in complex disease (reviewed in [20]). Pathway analyses of genes subject to altered DNA methylation identified autoimmune disease-relevant networks of genes including ‘immunological disease’, ‘cellular growth and proliferation’, and ‘antigen presentation’.

Four case samples used in the HM27 analysis had prior exposure to the commonly used DMARD, MTX. The anti-inflammatory properties of MTX are yet to be fully understood, but may include inhibition of T cell activation, induction of T cell apoptosis, and/or alteration of expression of cytokines [21]. Importantly for DNA methylation, MTX is an anti-folate agent. Folates are micronutrients essential for one-carbon metabolism, a process of methyl group transfer essential for the specific methylation of cytosine nucleotides within CpG sites [3]. Thus, it is reasonable to hypothesise that MTX may impact the T cell methylome in exposed cases. Whilst we cannot, from our data, provide conclusive evidence in support of this hypothesis, removal of MTX-exposed cases from the array analysis did have a striking impact on the outcome: the number of differentially methylated genes was reduced from 145 to 11; and none of the genes from the first- and second-tier analyses overlapped. While it remains possible that the contrasting outcomes were due to other factors, for example, the MTX-exposed cases may have been in other ways clinically distinct from the MTX-naive cases, our data does suggest a *potential* impact of MTX on DNA methylation. However, we found no evidence to suggest that this impact is genome-wide since mean β values across the >25,000 HM27 probes analysed was not different between exposed and unexposed cases (t-test p = 0.85). Unfortunately our sample size was insufficient for a meaningful locus-by-locus comparison of MTX-exposed and -naive case samples within this study, but overall our work suggests that data derived from MTX-exposed samples should be treated with caution in epigenome-wide association studies. Further investigation of this issue using a larger sample of MTX naive and exposed individuals is warranted, especially in light of recently published rheumatoid arthritis methylome data derived from case samples of mixed MTX exposure status [22].

Pathway analysis based on the 11 MTX-naive differentially methylated genes defined a network related to ‘cellular growth and proliferation, haematological system development and function, hematopoiesis’ within which nine of the 10 characterised genes were placed. Central to this network is the pro-inflammatory cytokine TNF. TNF initiates a pro-inflammatory programme of events associated with autoimmune and inflammatory diseases [23], including JIA [24]. TNF blockers have been used for some time as a treatment for JIA [25]. Therefore, aberrant methylation of a network of differentially methylated genes in T cells of children with JIA may result in the promotion of a disease-relevant inflammatory cascade.

Of the MTX-naive differentially methylated loci, *IL32* stands out as an interesting finding. IL32 is a pro-inflammatory cytokine shown to be a potent inducer of TNF. TNF also induces IL32 expression and the two molecules form an important autoinflammatory loop, as demonstrated in RA synovial cells [26]. Mice overexpressing IL32 show higher serum concentrations of TNF, and exacerbation of collagen-induced arthritis [27]. In our study, specific *IL32* CpGs measured by both genome-scale and locus specific analyses were seen to be less methylated in cases than in controls across two distinct sample sets, suggesting higher levels of expression. A limitation of our study is that we did not collect CD4+ T cell RNA to measure gene expression, although evidence does exist to suggest *IL32* expression is dependent on methylation [28]. Thus, further work will be required to assess the relationship between *IL32* DNA methylation and *IL32* gene expression in JIA.

The locus-specific replication data for *MRPL28* is also interesting. Although *MRPL28* was not seen to be significantly differentially methylated in the MTX-naive array analysis (FDR p = 0.47), significantly reduced methylation in cases relative to matched controls, (Δβ values in excess of 0.2), was found for pairs containing both MTX-exposed and -naive cases across discovery and replication samples. *MRPL28* encodes mitochondrial ribosomal protein L28, the function of which is poorly described. It is therefore difficult to speculate as to how reduced *MRPL28* methylation might increase JIA risk, although mitochondrial dysfunction has been associated with autoimmune disease [29]. Again, further work will be required to more fully interpret these findings.

An important issue that needs to be addressed in epigenetic research such as that presented here, is whether or not any identified disease-associated changes to the epigenome precede disease onset, or result from the disease process. Identification of aberrant DNA methylation that precedes disease suggests that such changes may contribute to risk of developing disease, and could therefore act as biomarkers to identify at-risk individuals and contribute to our understanding of the genomic underpinnings of causality. However, for relatively rare complex diseases like JIA, it is very difficult to prospectively collect cases, since the size of the prospective cohort necessary to identify sufficient cases would be very large. One approach to the problem is to retrospectively obtain diagnostic biospecimens collected routinely at birth, such as neonatal dried blood spots. Methods have now been developed to measure locus-specific DNA methylation in dried blood spots [30], and in the future this may well extend to the use of these specimens on a genome-scale. The CLARITY Biobank now encompasses neonatal dried blood spots from recruited cases and controls, and is shortly commencing longitudinal biospecimen collection for cases across the disease course. Thus, a future important research direction will be to examine changing methylation levels periodically from birth through to disease resolution.

## Conclusions

We have demonstrated genome-scale differences in T cell DNA methylation profiles in children with JIA compared to age- and sex-matched healthy controls. Our data suggests a potential impact of MTX treatment on the methylome, therefore selecting DMARD-naive biospecimens for future epigenetic research of this kind may be important. Finally, in two groups of case-control pairs across two different analysis platforms, methylation at *MRPL28* and *IL32* appears reduced in the T cells of children with JIA, and these findings were robust to MTX exposure. Other genes highlighted by the genome-scale analyses require further exploration. However, our data provides strong impetus to further consider the role of DNA methylation in JIA risk and in disease course, at higher genome-scale resolution and at specific loci, in larger sample sizes.

## Methods

### Participant recruitment and selection

Cases and controls were recruited as part of CLARITY (ChiLdhood Arthritis Risk factor Identification sTudY), an ongoing study of the genetic and environmental risk factors of JIA. Recruitment is ongoing, and commenced in 2008 at the Royal Children’s Hospital (RCH), Melbourne, Victoria Australia. Cases were defined as children meeting the International League of Associations for Rheumatology (ILAR) criteria [31] for the diagnosis of JIA. Cases were considered incident if they were recruited to the study within six months of initial diagnosis to allow for definition of ILAR subtype. Controls were otherwise well children ≤16 years admitted for minor surgical procedures. All protocols were approved by the RCH Human Research Ethics Committee.

For the genome-scale methylation array analysis, 14 cases (mean age 9.1 yrs, SD 4.2 yrs; 71.4% female; 10 oligoarticular, 4 polyarticular), were selected. The high percentage of female case samples reflects the predominance of females amongst those with oligoarticular and polyarticular JIA in the population. Cases were then matched to a control by age and sex. The basic characteristics of each ‘discovery’ case-control pair is shown in Additional file 1: Table S2 (pair numbers 1-14). All cases had active disease, and all were incident with the exception of cases in pairs 4 and 10.

An additional set of 12 incident cases (mean age 7.2 yrs, SD 4.2 yrs; 75.0% female; 9 oligoarticular, 3 polyarticular) and matched controls were selected in a similar fashion for replication of array-detected differentially methylated loci using the Sequenom MassARRAY platform. The basic characteristics of each ‘replication’ case-control pair is shown in Additional file 1: Table S2 (pair numbers 15-26).

### T cell DNA isolation and genome-scale methylation analysis

Each participant provided a 9ml peripheral blood sample. Plasma was removed within two hours of collection and stored. PBMCs were isolated using standard Ficoll gradient procedures within 24 hours of collection, and stored in liquid nitrogen. Total CD3+ CD4+ T cells were positively selected from the PBMC population using flow cytometry (Becton Dickinson (San Jose, CA, USA) antibodies, cat# 340440, 347327). T cell purity was very high (typically ~99%) following cell sorting. DNA was extracted using the Flexigene DNA extraction kit (Qiagen) and bisulphite converted using the MethylEasy Xceed (Human Genetic Signatures, Randwick, NSW, Australia) kit according to manufacturer’s instructions. Converted DNA was then applied to the Illumina Infinium HumanMethylation27 BeadChip arrays (HM27) (ServiceXS, The Netherlands). These arrays measure methylation at an average of 2 CpG sites in the promoter regions of 14,475 genes and 110 miRNA promoters (27,578 sites tested in all). DNA from each case and its matched control was hybridised to the same array chip to avoid confounding of results by chip variability. DNA samples from one case-control pair (pair number 13) were applied twice to the same chip as technical replicates.

### Genome-scale methylation data analysis

All analyses were carried out using the statistical programming language R ( http://www.r-project.org) (version 2.12.0) using packages from the Bioconductor project [32]. Data quality was confirmed using *arrayQualityMetrics*[33]*,* which produces a number of diagnostic plots to assess reproducibility, identifies apparent outlier arrays and computes measures of the signal-to-noise ratio.

Probes considered to overlap SNPs [34] were removed, along with those on the X and Y chromosomes, reducing the number of probes from 27,578 to 26,422. The *lumi* package [35,36] was used to calculate the log2 ratios for methylated probe intensity to unmethylated probe intensity (M-values). These values underwent colour-adjustment, background correction and ssn normalization. All probes failing a detection p-value cut-off of < 0.01 were removed. This reduced the number of probes available to 25,303 probes (13,462 unique genes). These probes were subsequently passed to *limma*[37] for differential methylation analysis. Array weights were calculated, thereby estimating relative quality weights for each array [38]. A linear model was fitted testing for the difference between cases and controls. P-values were adjusted for multiple testing using the Benjamini and Hochberg method [39]. M-values were converted to β-values (equivalent to percent of alleles methylated at each site) and subsequently used to calculate the correlation between the technical replicates.

### Gene Ontology and Pathway Analyses

Testing was carried out for the association of Gene Ontology (GO) terms in the list of significantly differentially methylated genes using the Bioconductor package GOstat [40]. Specifically an analysis was carried out to look for overrepresentation of GO terms using a hypergeometric test. Ingenuity Pathway Analysis (IPA) software (Ingenuity Systems, Redwood City, CA, USA) was used to investigate pathways and relationships between differentially methylated genes. The full list of gene loci measured by the HM27 was used as the reference set in these analyses.

### Validation of case-control methylation differences using the Sequenom MassARRAY Epityper platform

Validation of array-detected methylation differences between cases and controls at chosen loci was performed using the Sequenom (San Diego, CA, USA) MassARRAY platform as previously described [41]. Assays were designed using the Epidesigner software ( http://www.epidesigner.com) to capture methylation at CpG sites surrounding the HM27 array probe, with the exact HM27 CpG site measured where possible (Additional file 1: Figures S3 and S4). Data points were removed where differences in duplicate beta values exceeded 0.2, otherwise the average was taken. Stata v11 (StataCorp, College Station, TEX, USA) was used to perform HM27-Sequenom data correlation analyses and conditional logistic regression analyses (taking into account the matched-pair design) to examine the differences in methylation β values (converted to % methylation by multiplying β values by 100) between cases and controls.

## Competing interests

The authors declare that they have no competing interests.

## Authors’ contributions

JE, RS, JC, JM, RC designed the study. JM, JA, RA oversaw recruitment and advised on clinical aspects of the study. RC, JEJ carried out the laboratory work. LG, JE, RS, JC, ALP carried out statistical data analysis and interpretation. All authors contributed to manuscript writing and approved the final manuscript.

## Supplementary Material

Additional file 1 **Figure S1.** Correlation in betas between technical replicates. **Table S1.** Alphabetical list of genes associated with probes identified as significantly differentially methylated between cases and controls. **Figure S2.** Heatmap of significantly differentially methylated loci between MTX-naïve JIA cases and controls. **Figure S3.** Location of *MRPL28* CpG dinucleotides measured by the Sequenom Epityper assay. **Figure S4.** Location of *IL32* CpG dinucleotides measured by the Sequenom Epityper assay. **Table S2.** Basic characteristics of the age- and sex-matched case-control pairs. **Table S3.** Beta values for *MRPL28* Sequenom EpiTYPER assay units, in comparison to beta values from the significantly differentially methylated *MRPL28* HM27 array probe. **Table S4.** Beta values for *IL32* Sequenom EpiTYPER assay units, in comparison to beta values from the significantly differentially methylated *IL32* HM27 array probe. Click here for file

## References

[B1] Ellis JA Munro JE Ponsonby AL Possible environmental determinants of juvenile idiopathic arthritis Rheumatology (Oxford) 2010 49 411 425 10.1093/rheumatology/kep383 19965974

[B2] Bird A Perceptions of epigenetics Nature 2007 447 396 398 10.1038/nature05913 17522671

[B3] Foley DL Craig JM Morley R Olsson CA Dwyer T Smith K Saffery R Prospects for epigenetic epidemiology Am J Epidemiol 2009 169 389 400 19139055 10.1093/aje/kwn380 PMC3290967

[B4] Waterland RA Jirtle RL Transposable elements: targets for early nutritional effects on epigenetic gene regulation Mol Cell Biol 2003 23 5293 5300 10.1128/MCB.23.15.5293-5300.2003 12861015 PMC165709

[B5] Bell JT Saffery R The value of twins in epigenetic epidemiology Int J Epidemiol 2012 41 140 150 10.1093/ije/dyr179 22253312

[B6] Meda F Folci M Baccarelli A Selmi C The epigenetics of autoimmunity Cell Mol Immunol 2011 8 226 236 10.1038/cmi.2010.78 21278766 PMC3093958

[B7] Janson PC Winerdal ME Winqvist O At the crossroads of T helper lineage commitment-Epigenetics points the way Biochim Biophys Acta 2009 1790 906 919 10.1016/j.bbagen.2008.12.003 19162128

[B8] Wells AD New insights into the molecular basis of T cell anergy: anergy factors, avoidance sensors, and epigenetic imprinting J Immunol 2009 182 7331 7341 10.4049/jimmunol.0803917 19494254

[B9] Balada E Ordi-Ros J Vilardell-Tarres M DNA methylation and systemic lupus erythematosus Ann N Y Acad Sci 2007 1108 127 136 10.1196/annals.1422.015 17893979

[B10] Javierre BM Fernandez AF Richter J Al-Shahrour F Martin-Subero JI Rodriguez-Ubreva J Berdasco M Fraga MF O'Hanlon TP Rider LG Changes in the pattern of DNA methylation associate with twin discordance in systemic lupus erythematosus Genome Res 2010 20 170 179 10.1101/gr.100289.109 20028698 PMC2813473

[B11] Liu CC Fang TJ Ou TT Wu CC Li RN Lin YC Lin CH Tsai WC Liu HW Yen JH Global DNA methylation, DNMT1, and MBD2 in patients with rheumatoid arthritis Immunol Lett 2011 135 96 99 10.1016/j.imlet.2010.10.003 20937307

[B12] Richardson B Scheinbart L Strahler J Gross L Hanash S Johnson M Evidence for impaired T cell DNA methylation in systemic lupus erythematosus and rheumatoid arthritis Arthritis Rheum 1990 33 1665 1673 10.1002/art.1780331109 2242063

[B13] Karouzakis E Gay RE Michel BA Gay S Neidhart M DNA hypomethylation in rheumatoid arthritis synovial fibroblasts Arthritis Rheum 2009 60 3613 3622 10.1002/art.25018 19950268

[B14] Nile CJ Read RC Akil M Duff GW Wilson AG Methylation status of a single CpG site in the IL6 promoter is related to IL6 messenger RNA levels and rheumatoid arthritis Arthritis Rheum 2008 58 2686 2693 10.1002/art.23758 18759290

[B15] Takami N Osawa K Miura Y Komai K Taniguchi M Shiraishi M Sato K Iguchi T Shiozawa K Hashiramoto A Shiozawa S Hypermethylated promoter region of DR3, the death receptor 3 gene, in rheumatoid arthritis synovial cells Arthritis Rheum 2006 54 779 787 10.1002/art.21637 16508942

[B16] De Santis M Selmi C The therapeutic potential of epigenetics in autoimmune diseases Clin Rev Allergy Immunol 2012 42 92 101 10.1007/s12016-011-8293-8 22161696

[B17] Hollenbach JA Thompson SD Bugawan TL Ryan M Sudman M Marion M Langefeld CD Thomson G Erlich HA Glass DN Juvenile idiopathic arthritis and HLA class I and class II interactions and age-at-onset effects Arthritis Rheum 2010 62 1781 1791 10.1002/art.27424 20191588 PMC2917207

[B18] Dam AH Koscinski I Kremer JA Moutou C Jaeger AS Oudakker AR Tournaye H Charlet N Lagier-Tourenne C van Bokhoven H Viville S Homozygous mutation in SPATA16 is associated with male infertility in human globozoospermia Am J Hum Genet 2007 81 813 820 10.1086/521314 17847006 PMC2227931

[B19] Rajagopalan PT Zhang Z McCourt L Dwyer M Benkovic SJ Hammes GG Interaction of dihydrofolate reductase with methotrexate: ensemble and single-molecule kinetics Proc Natl Acad Sci U S A 2002 99 13481 13486 10.1073/pnas.172501499 12359872 PMC129699

[B20] Rakyan VK Beyan H Down TA Hawa MI Maslau S Aden D Daunay A Busato F Mein CA Manfras B Identification of type 1 diabetes-associated DNA methylation variable positions that precede disease diagnosis PLoS Genet 2011 7 e1002300 10.1371/journal.pgen.1002300 21980303 PMC3183089

[B21] Aune ZT Spurlock CF 3rd Aune ZT Tossberg JT Collins PL Aune JP Huston JW 3rd Crooke PS Olsen NJ Aune TM Increased sensitivity to apoptosis induced by methotrexate is mediated by JNK Arthritis Rheum 2011 63 2606 2616 10.1002/art.30457 21618198 PMC3165146

[B22] Nakano K Whitaker JW Boyle DL Wang W Firestein GS DNA methylome signature in rheumatoid arthritis Ann Rheum Dis 2012 10.1136/annrheumdis-2012-201526 PMC3549371 22736089

[B23] Croft M The role of TNF superfamily members in T-cell function and diseases Nat Rev Immunol 2009 9 271 285 10.1038/nri2526 19319144 PMC2737409

[B24] de Jager W Hoppenreijs EP Wulffraat NM Wedderburn LR Kuis W Prakken BJ Blood and synovial fluid cytokine signatures in patients with juvenile idiopathic arthritis: a cross-sectional study Ann Rheum Dis 2007 66 589 598 10.1136/ard.2006.061853 17170049 PMC1954617

[B25] Hashkes PJ Uziel Y Laxer RM The safety profile of biologic therapies for juvenile idiopathic arthritis Nat Rev Rheumatol 2010 6 561 571 10.1038/nrrheum.2010.142 20808294

[B26] Heinhuis B Koenders MI van Riel PL van de Loo FA Dinarello CA Netea MG van den Berg WB Joosten LA Tumour necrosis factor alpha-driven IL-32 expression in rheumatoid arthritis synovial tissue amplifies an inflammatory cascade Ann Rheum Dis 2011 70 660 667 10.1136/ard.2010.139196 21187297

[B27] Shoda H Fujio K Yamaguchi Y Okamoto A Sawada T Kochi Y Yamamoto K Interactions between IL-32 and tumor necrosis factor alpha contribute to the exacerbation of immune-inflammatory diseases Arthritis Res Ther 2006 8 R166 10.1186/ar2074 17078892 PMC1794509

[B28] Li W Sun W Liu L Yang F Li Y Chen Y Fang J Zhang W Wu J Zhu Y IL-32: a host proinflammatory factor against influenza viral replication is upregulated by aberrant epigenetic modifications during influenza A virus infection J Immunol 2010 185 5056 5065 10.4049/jimmunol.0902667 20889550

[B29] Vyshkina T Sylvester A Sadiq S Bonilla E Canter JA Perl A Kalman B Association of common mitochondrial DNA variants with multiple sclerosis and systemic lupus erythematosus Clin Immunol 2008 129 31 35 10.1016/j.clim.2008.07.011 18708297 PMC2567049

[B30] Wong N Morley R Saffery R Craig J Archived Guthrie blood spots as a novel source for quantitative DNA methylation analysis Biotechniques 2008 45 423 424 426, 428 passim 10.2144/000112945 18855769

[B31] Petty RE Southwood TR Manners P Baum J Glass DN Goldenberg J He X Maldonado-Cocco J Orozco-Alcala J Prieur AM International League of Associations for Rheumatology classification of juvenile idiopathic arthritis: second revision, Edmonton, 2001 J Rheumatol 2004 31 390 392 14760812

[B32] Gentleman RC Carey VJ Bates DM Bolstad B Dettling M Dudoit S Ellis B Gautier L Ge Y Gentry J Bioconductor: open software development for computational biology and bioinformatics Genome Biol 2004 5 R80 10.1186/gb-2004-5-10-r80 15461798 PMC545600

[B33] Kauffmann A Gentleman R Huber W arrayQualityMetrics--a bioconductor package for quality assessment of microarray data Bioinformatics 2009 25 415 416 10.1093/bioinformatics/btn647 19106121 PMC2639074

[B34] Chen YA Choufani S Ferreira JC Grafodatskaya D Butcher DT Weksberg R Sequence overlap between autosomal and sex-linked probes on the Illumina HumanMethylation27 microarray Genomics 2011 97 214 222 10.1016/j.ygeno.2010.12.004 21211562

[B35] Du P Kibbe WA Lin SM lumi: a pipeline for processing Illumina microarray Bioinformatics 2008 24 1547 1548 10.1093/bioinformatics/btn224 18467348

[B36] Du P Zhang X Huang CC Jafari N Kibbe WA Hou L Lin SM Comparison of Beta-value and M-value methods for quantifying methylation levels by microarray analysis BMC Bioinforma 2010 11 587 10.1186/1471-2105-11-587 PMC3012676 21118553

[B37] Smyth G Gentleman R, Carey V, Dudoit S, Irizarry R, Huber W Limma: linear models for microarray data. Bioinformatics and Computational Biology Solutions using R and Bioconductor 2005 New York: Springer 397 420

[B38] Ritchie ME Diyagama D Neilson J van Laar R Dobrovic A Holloway A Smyth GK Empirical array quality weights in the analysis of microarray data BMC Bioinforma 2006 7 261 10.1186/1471-2105-7-261 PMC1564422 16712727

[B39] Benjamini Y Hochberg Y Controlling the false discovery rate: a practical and powerful approach to multiple testing J R Stat Soc B 1995 57 289 300

[B40] Falcon S Gentleman R Using GOstats to test gene lists for GO term association Bioinformatics 2007 23 257 258 10.1093/bioinformatics/btl567 17098774

[B41] Ollikainen M Smith KR Joo EJ Ng HK Andronikos R Novakovic B Abdul Aziz NK Carlin JB Morley R Saffery R Craig JM DNA methylation analysis of multiple tissues from newborn twins reveals both genetic and intrauterine components to variation in the human neonatal epigenome Hum Mol Genet 2010 19 4176 4188 10.1093/hmg/ddq336 20699328

