# Supplementary material for: Genome-scale case-control analysis of CD4+ T-cell DNA methylation in juvenile idiopathic arthritis reveals potential targets involved in disease
Source: Clin Epigenetics. 2012 Nov 13;4(1):20. doi: 10.1186/1868-7083-4-20 (PMC3536591; doi:10.1186/1868-7083-4-20)
Supplement: Additional file 1 — Figure S1. Correlation in betas between technical replicates. Table S1. Alphabetical list of genes associated with probes identified as significantly differentially methylated between cases and controls. Figure S2. Heatmap of significantly differentially methylated loci between MTX-naïve JIA cases and controls. Figure S3. Location of MRPL28 CpG dinucleotides measured by the Sequenom Epityper assay. Figure S4. Location of IL32 CpG dinucleotides measured by the Sequenom Epityper assay. Table S2. Basic characteristics of the age- and sex-matched case-control pairs. Table S3. Beta values for MRPL28 Sequenom EpiTYPER assay units, in comparison to beta values from the significantly differentially methylated MRPL28 HM27 array probe. Table S4. Beta values for IL32 Sequenom EpiTYPER assay units, in comparison to beta values from the significantly differentially methylated IL32 HM27 array probe. [file 1868-7083-4-20-S1.pdf]

Supplementary Figure 1: Correlation in betas between technical replicates.

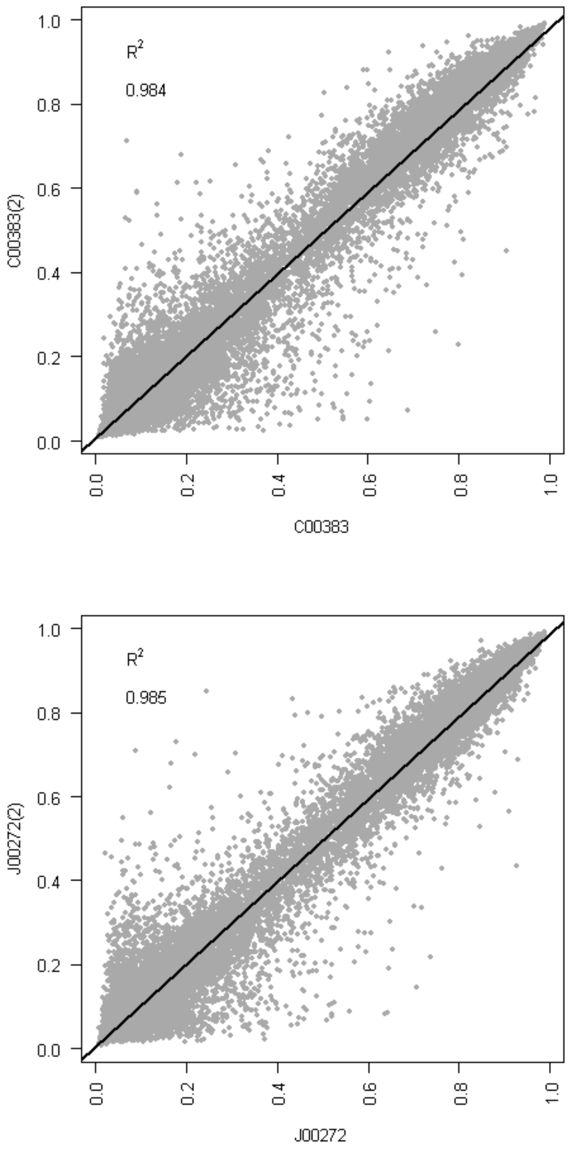

Supplementary Table 1: Alphabetical list of genes associated with probes identified as significantly differentially methylated between cases and controls.

GENE	P.Value	adj.P.Value*	B	Case relative to control	Median controls	Median cases	Difference
AKAP6	0.000216306	0.083714351	0.699780073	Higher	0.819	0.873	0.054
ALDOB	6.63849E-05	0.068594965	1.727680478	Higher	0.715	0.791	0.075
ANGEL2	0.00027652	0.08962433	0.485163328	Lower	0.106	0.082	0.024
APOE	0.000126617	0.080318586	1.16679729	Higher	0.829	0.861	0.032
ARFGEF1	0.000490418	0.095936966	-0.016377223	Lower	0.154	0.110	0.045
ARHGDIB	0.00022254	0.083714351	0.674965243	Lower	0.176	0.124	0.052
ARMC1	0.000331375	0.095936966	0.32686873	Lower	0.287	0.205	0.081
ART4	0.000480166	0.095936966	0.002131267	Higher	0.833	0.864	0.032
ASB4	0.000321289	0.095936966	0.353913712	Higher	0.803	0.877	0.074
ATP2B1	0.000505442	0.095936966	-0.042816174	Higher	0.761	0.824	0.063
ATP2B4	0.000424007	0.095936966	0.111080164	Lower	0.437	0.323	0.114
BAP1	0.000433075	0.095936966	0.092548349	Lower	0.196	0.157	0.038
BCDO2	0.000103549	0.080072606	1.341785717	Lower	0.109	0.086	0.024
BCKDHB	0.000446559	0.095936966	0.065692583	Lower	0.200	0.154	0.046
BLR1	0.00039704	0.095936966	0.168623889	Higher	0.523	0.600	0.078
C11orf49	0.000199824	0.083714351	0.768988887	Lower	0.179	0.140	0.039
C12orf26	0.00012176	0.080318586	1.200846154	Higher	0.828	0.867	0.039
C14orf8	0.000127645	0.080318586	1.159756402	Higher	0.819	0.865	0.046
C15orf15	0.000571684	0.099760877	-0.150736065	Lower	0.221	0.162	0.059
C18orf20	0.000395139	0.095936966	0.172826098	Higher	0.768	0.860	0.093
C19orf18	0.000241853	0.08499455	0.602255319	Higher	0.783	0.839	0.056
C6orf66	0.000273175	0.08962433	0.495806312	Lower	0.128	0.096	0.032
C7orf26	0.000182942	0.083714351	0.84602795	Higher	0.686	0.774	0.088
C8ORFK32	4.09043E-05	0.066658033	2.146409328	Higher	0.754	0.794	0.040
CA9	0.000472019	0.095936966	0.017122877	Higher	0.935	0.947	0.012
CARD8	0.000434989	0.095936966	0.088686498	Lower	0.135	0.088	0.047
CASP8	0.000348705	0.095936966	0.28226019	Lower	0.123	0.095	0.029
CAV3	0.00052839	0.098307717	-0.081721183	Higher	0.919	0.941	0.022

CBARA1	0.000256968	0.089069361	0.549273653	Lower	0.099	0.077	0.023
CBR4	0.00049384	0.095936966	-0.022468975	Lower	0.106	0.089	0.017
CCDC23	0.000508064	0.095936966	-0.047349657	Higher	0.703	0.767	0.064
CCKAR	0.000426096	0.095936966	0.106776349	Higher	0.887	0.907	0.020
CD53	0.000476737	0.095936966	0.00840975	Lower	0.171	0.136	0.035
CDS2	0.000381132	0.095936966	0.204425604	Higher	0.626	0.704	0.078
CEACAM3	0.000303238	0.094575911	0.404499515	Higher	0.746	0.815	0.069
CECR5	0.000534147	0.098653532	-0.091217994	Higher	0.661	0.730	0.069
CHD2	0.000455031	0.095936966	0.049231605	Lower	0.269	0.194	0.074
CLOCK	0.000110759	0.080072606	1.28324222	Higher	0.822	0.881	0.059
COL17A1	0.000270035	0.08962433	0.505915444	Higher	0.688	0.754	0.066
CPA4	0.000122594	0.080318586	1.194903134	Higher	0.668	0.764	0.097
CPT2	0.000233058	0.08499455	0.634620657	Lower	0.148	0.110	0.038
CRB1	0.000370595	0.095936966	0.228968726	Higher	0.885	0.921	0.036
CREG1	0.000387024	0.095936966	0.190994396	Lower	0.199	0.148	0.052
CYP4Z1	0.000342478	0.095936966	0.298029077	Higher	0.546	0.604	0.058
DCTN3	0.000387663	0.095936966	0.189549856	Lower	0.126	0.098	0.028
DDX18	0.00036628	0.095936966	0.239220057	Lower	0.095	0.060	0.035
DEFB129	0.000306494	0.094575911	0.395156672	Higher	0.684	0.755	0.071
DGKH	6.38106E-05	0.068594965	1.761945858	Lower	0.114	0.089	0.025
DNAH8	8.11778E-05	0.07008303	1.553221977	Higher	0.847	0.886	0.039
DOK2	0.000279821	0.08962433	0.474786482	Lower	0.207	0.147	0.060
ESR2	0.000206769	0.083714351	0.739158314	Lower	0.104	0.084	0.020
FAM91A1	7.12236E-05	0.068594965	1.66670021	Higher	0.692	0.755	0.063
FLJ10803	0.000393219	0.095936966	0.177092075	Lower	0.256	0.203	0.053
FLJ20581	2.65243E-05	0.066658033	2.519401371	Higher	0.877	0.924	0.047
FLJ36004	4.64768E-05	0.066658033	2.036142035	Higher	0.738	0.823	0.085
FLJ40142	0.000221324	0.083714351	0.679751903	Higher	0.856	0.885	0.029
FLJ44674	0.000176729	0.083714351	0.87617517	Higher	0.814	0.858	0.043
FXC1	0.000155055	0.083475942	0.990274457	Higher	0.743	0.812	0.069
GCET2	5.88214E-06	0.066658033	3.802565003	Lower	0.152	0.107	0.044
GLDN	0.000430652	0.095936966	0.097461736	Higher	0.810	0.848	0.039
GP2	0.000557386	0.09885596	-0.128537656	Higher	0.733	0.783	0.050
GPD2	0.000485084	0.095936966	-0.00679635	Higher	0.713	0.778	0.065
GRM5	3.35772E-05	0.066658033	2.316566914	Higher	0.780	0.846	0.066
GYPE	1.71608E-05	0.066658033	2.892676923	Higher	0.688	0.781	0.093

HINT1	0.000486927	0.095936966	-0.010117612	Lower	0.171	0.131	0.040
HKR1	0.000449784	0.095936966	0.059390999	Lower	0.385	0.319	0.066
HNMT	0.00055148	0.09885596	-0.119202919	Higher	0.786	0.847	0.060
HRSP12	0.0005018	0.095936966	-0.036479154	Lower	0.075	0.054	0.021
IL1RL1	0.000484402	0.095936966	-0.005563891	Higher	0.698	0.785	0.087
ILDR1	0.000144504	0.081253071	1.051698105	Higher	0.855	0.889	0.034
ITGA6	0.000506104	0.095936966	-0.043962533	Lower	0.144	0.114	0.030
KIAA0196	0.00019609	0.083714351	0.785456614	Higher	0.772	0.849	0.077
KIAA0746	0.00011047	0.080072606	1.285519142	Lower	0.218	0.144	0.074
KRT10	0.000517814	0.09705368	-0.064004651	Higher	0.721	0.799	0.078
KRT25A	0.00021474	0.083714351	0.706129139	Higher	0.833	0.885	0.052
LCN12	0.000400885	0.095936966	0.160184714	Higher	0.907	0.922	0.015
LECT2	0.000339955	0.095936966	0.304501457	Higher	0.755	0.823	0.068
LILRB4	3.38863E-05	0.066658033	2.308676718	Higher	0.823	0.866	0.043
LOC253012	0.000540447	0.09885596	-0.101492465	Higher	0.887	0.917	0.029
LOC90826	1.21834E-05	0.066658033	3.185062762	Lower	0.142	0.091	0.051
LONRF2	0.000206915	0.083714351	0.738544738	Higher	0.647	0.711	0.065
LPO	0.000202473	0.083714351	0.757490996	Higher	0.761	0.803	0.043
LRRC46	0.00013367	0.080318586	1.119591765	Higher	0.653	0.753	0.101
LY9	0.000109007	0.080072606	1.297116731	Lower	0.153	0.117	0.036
MCM5	0.000178767	0.083714351	0.866174012	Higher	0.927	0.947	0.021
MEF2C	3.57177E-05	0.066658033	2.26332763	Lower	0.075	0.056	0.019
MGC33839	0.00035497	0.095936966	0.26667493	Higher	0.817	0.846	0.030
MGC40222	0.000241018	0.08499455	0.605278938	Higher	0.865	0.896	0.031
MIA2	0.000385632	0.095936966	0.194150061	Higher	0.749	0.821	0.072
MKS1	0.000477723	0.095936966	0.00659922	Higher	0.691	0.807	0.116
MRPL28	4.03006E-05	0.066658033	2.159236828	Lower	0.875	0.685	0.190
MTRF1	0.000439661	0.095936966	0.079329301	Lower	0.178	0.131	0.046
NDST4	0.000136283	0.080318586	1.102732092	Higher	0.825	0.861	0.036
NDUFV1	0.00013654	0.080318586	1.101086123	Higher	0.871	0.904	0.033
NR1D1	0.000208204	0.083714351	0.73311983	Lower	0.219	0.160	0.059
NR1I2	3.37791E-05	0.066658033	2.31140486	Higher	0.842	0.890	0.047
OTX2	0.000452795	0.095936966	0.05354545	Lower	0.243	0.197	0.046
PCYT2	0.000271071	0.08962433	0.502567167	Higher	0.840	0.867	0.028
PDIA3	0.000562592	0.09885596	-0.136684845	Lower	0.098	0.071	0.028
PLUNC	5.42899E-05	0.068594965	1.901817742	Higher	0.798	0.859	0.061

PRH1	2.83945E-05	0.066658033	2.46083631	Higher	0.813	0.875	0.062
PRH2	0.000214014	0.083714351	0.70908677	Higher	0.808	0.841	0.033
PRPF8	0.000498552	0.095936966	-0.030789517	Lower	0.131	0.103	0.028
PSMB5	0.000241048	0.08499455	0.605169788	Lower	0.135	0.097	0.038
PSMC5	0.00019204	0.083714351	0.803672493	Higher	0.795	0.868	0.073
PYGM	0.000346549	0.095936966	0.287687714	Higher	0.814	0.865	0.050
RAB32	0.000440251	0.095936966	0.078155013	Higher	0.772	0.817	0.045
RAD51L3	4.58085E-05	0.066658033	2.048653596	Lower	0.277	0.202	0.075
RBM18	0.000213842	0.083714351	0.70978614	Higher	0.817	0.858	0.041
RBP3	0.000166416	0.083714351	0.928619929	Higher	0.758	0.848	0.090
RETN	0.00055767	0.09885596	-0.12898426	Higher	0.712	0.791	0.079
Rgr	4.12023E-06	0.066658033	4.102342388	Higher	0.894	0.931	0.037
RHEB	0.000380226	0.095936966	0.206508863	Lower	0.111	0.084	0.026
RPL34	0.000224976	0.083714351	0.665454231	Lower	0.161	0.117	0.043
RPS7	0.000215054	0.083714351	0.704850796	Higher	0.076	0.103	0.028
SCML4	0.000443466	0.095936966	0.071780197	Higher	0.831	0.863	0.031
SCOC	4.74191E-05	0.066658033	2.018801341	Higher	0.765	0.825	0.060
SELL	8.30926E-05	0.07008303	1.532987087	Lower	0.168	0.118	0.050
SERINC3	0.000441112	0.095936966	0.076442643	Lower	0.168	0.109	0.059
SLAMF7	0.000560297	0.09885596	-0.133102327	Higher	0.741	0.794	0.053
SLC13A3	2.34849E-05	0.066658033	2.623903316	Higher	0.712	0.784	0.073
SLC1A6	0.000465285	0.095936966	0.029709668	Higher	0.812	0.871	0.059
SLC22A6	7.59064E-05	0.068594965	1.611478488	Higher	0.646	0.751	0.105
SLC4A1	0.000130531	0.080318586	1.140289798	Higher	0.773	0.835	0.062
SPA17	7.46543E-05	0.068594965	1.62590524	Lower	0.194	0.140	0.054
SPATA16	9.95298E-05	0.080072606	1.376197121	Higher	0.844	0.904	0.060
SPOP	6.32517E-05	0.068594965	1.769566229	Lower	0.216	0.178	0.038
TAS2R60	0.000549771	0.09885596	-0.116483034	Higher	0.807	0.849	0.042
TBC1D7	0.000149293	0.082120933	1.023285487	Higher	0.777	0.834	0.057
TDRD1	0.000139668	0.080318586	1.081357048	Higher	0.746	0.792	0.046
TEKT3	5.67896E-05	0.068594965	1.862870314	Higher	0.303	0.409	0.106
TFB1M	7.54951E-05	0.068594965	1.616191213	Higher	0.748	0.821	0.073
THEM5	0.000403019	0.095936966	0.155537503	Higher	0.744	0.794	0.050
TIMM8B	1.56402E-05	0.066658033	2.971983671	Lower	0.124	0.079	0.045
TLR6	0.000188279	0.083714351	0.820934811	Higher	0.834	0.876	0.042
TMCO5	0.00032289	0.095936966	0.349566165	Higher	0.774	0.847	0.074

TP53BP1	0.000272418	0.08962433	0.498233089	Lower	0.089	0.061	0.028
TRAM1L1	4.50755E-05	0.066658033	2.062585417	Lower	0.224	0.142	0.083
TRPV5	0.000504023	0.095936966	-0.04035213	Higher	0.879	0.920	0.041
TSC22D2	0.0003061	0.094575911	0.396282629	Lower	0.037	0.031	0.006
YWHAB	7.39695E-05	0.068594965	1.633897346	Higher	0.870	0.910	0.040
ZNF217	0.000458685	0.095936966	0.042224996	Lower	0.204	0.114	0.090
ZNF306	0.000444446	0.095936966	0.069848113	Lower	0.190	0.147	0.043
ZNF536	0.000427091	0.095936966	0.104734155	Higher	0.605	0.677	0.072
ZNF678	0.000166443	0.083714351	0.928480869	Higher	0.792	0.855	0.063

* P value following adjustment for false discovery rate (FDR)

B = log-odds that the probe is differentially methylated

Supplementary Figure 2. Heatmap of significantly differentially methylated loci between MTX-naïve JIA cases and controls. Black = case, Grey = control. 'Value' = percent methylation.

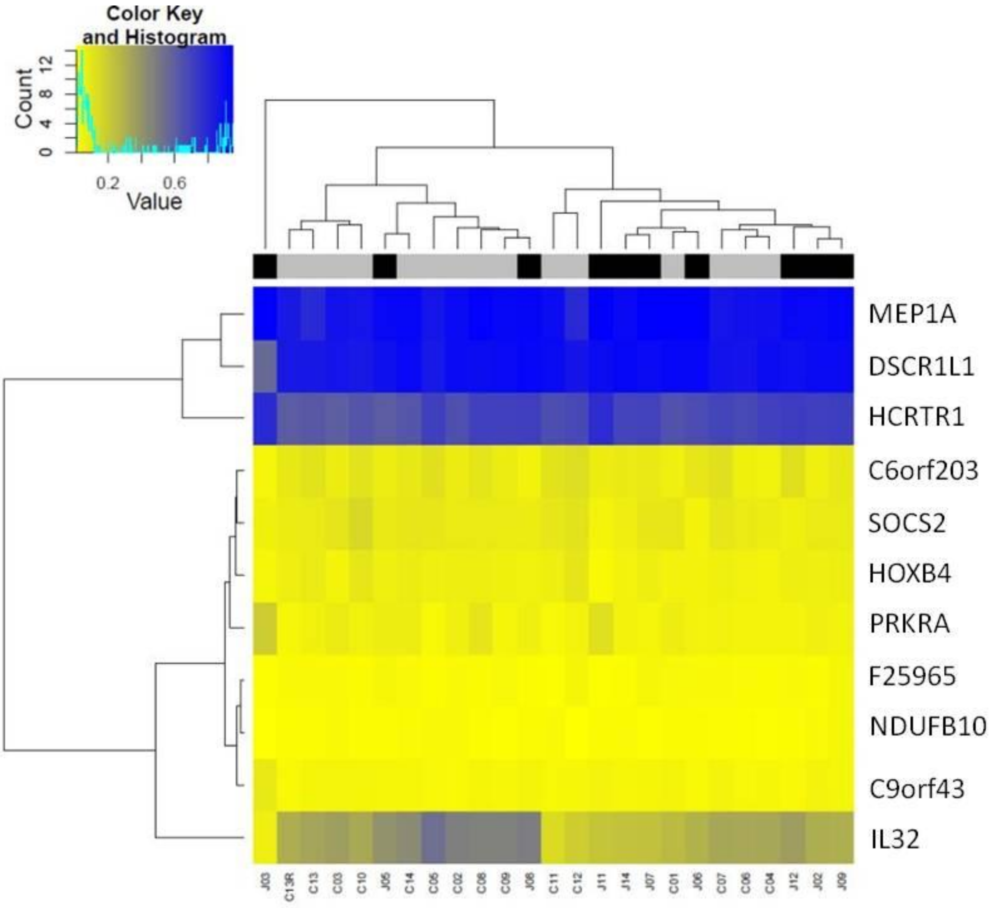

Supplementary Figure 3: Location of *MRPL28* CpG dinucleotides measured by the Sequenom Epityper assay. Assay unit denoted in red includes the significant HM27 CpG probe site.

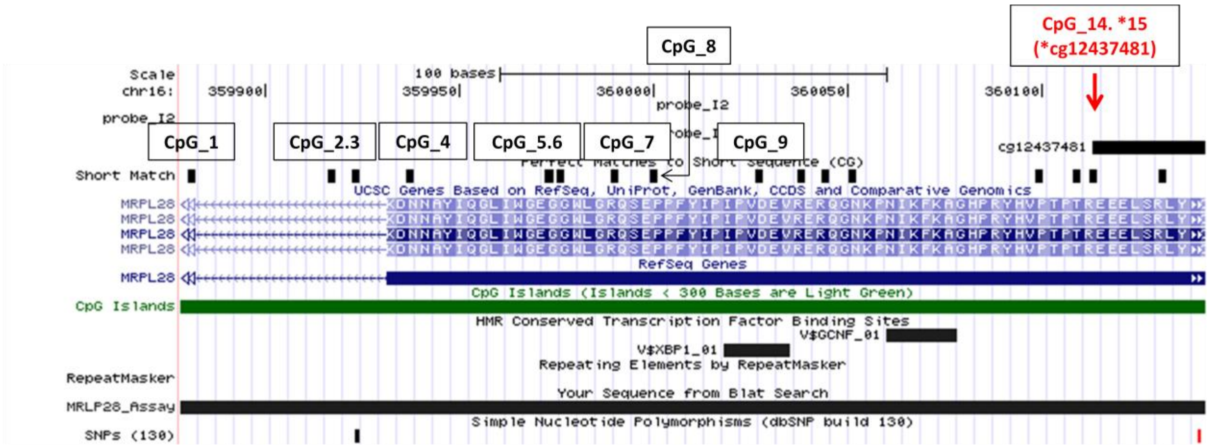

Supplementary Figure 4: Location of *IL32* CpG dinucleotides measured by the Sequenom Epityper assay. CpGs were measured using two separate assays, denoted here as ‘IL32_a’ and ‘IL32_b’. Assay unit denoted in red and asterisked includes the significant HM27 CpG probe site.

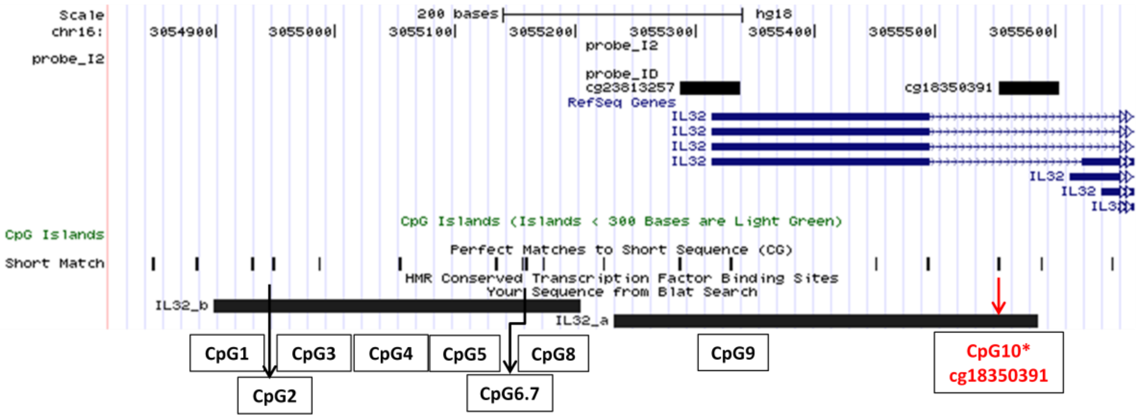

Supplementary Table 2: Basic characteristics of the age- and sex-matched case-control pairs. Cases of pairs in **bold** were exposed to MTX prior to biospecimen collection.

Case-control pair #	Age at recruitment	Age at diagnosis*	Sex	JIA subtype
Discovery (array):				
**01**	**1**	**1**	**F**	**polyarticular**
02	2	2	F	oligoarticular
03	5	5	F	polyarticular
**04**	**6**	**2**	**F**	**oligoarticular**
05	7	6	F	oligoarticular
06	9	9	F	oligoarticular
07	11	11	F	oligoarticular
08	12	11	M	oligoarticular
09	12	12	M	oligoarticular
**10**	**12**	**7**	**F**	**polyarticular**
11	11	11	M	polyarticular
12	12	11	F	oligoarticular
**13**	**13**	**13**	**F**	**oligoarticular**
14	14	14	M	oligoarticular
Replication:				
15	1	1	M	oligoarticular
16	3	3	F	oligoarticular
17	6	6	F	oligoarticular
18	8	8	F	oligoarticular
**19**	**11**	**10**	**M**	**polyarticular**
20	12	12	F	oligoarticular
21	13	13	F	oligoarticular
22	6	6	F	oligoarticular
23	10	10	M	oligoarticular
24	11	11	F	polyarticular
25	2	2	F	oligoarticular
26	3	3	F	polyarticular

* Case only

Supplementary Table 3: Beta values for *MRPL28* Sequenom EpiTYPER assay units, in comparison to beta values from the significantly differentially methylated *MRPL28* HM27 array probe. The array probe could not be captured in isolation by the Sequenom assay, but forms part of the assay unit labeled CpG_14.15. N/A = not assayed. "-" = missing data (did not pass quality control).

		Sequenom assay unit								HM27 array probe
Pair #	sample type	CpG_1	CpG_2.3	CpG_4	CpG_5.6	CpG_7	CpG_8	CpG_9	CpG_14.15	cg12437481
'array' pairs										
1	case	0.44	0.95	0.65	0.95	0.90	0.96	0.61	0.81	0.79
	control	0.46	0.95	0.46	0.95	0.90	0.85	0.43	0.73	0.66
2	case	0.43	0.96	0.76	0.95	0.91	0.90	0.67	0.82	0.88
	control	0.47	0.96	0.86	0.96	0.95	0.97	0.71	0.90	0.91
3	case	0.51	0.93	0.35	0.64	0.53	0.49	0.16	0.38	0.34
	control	0.43	0.97	0.73	0.95	0.94	0.96	0.61	0.82	0.84
4	case	0.49	0.93	0.59	0.92	0.84	0.91	0.57	0.68	0.58
	control	0.45	0.93	0.73	0.92	0.92	0.91	0.61	0.82	0.77
6	case	0.48	0.93	0.56	0.78	0.71	0.76	0.51	0.62	0.42
	control	0.47	0.96	0.82	0.96	0.95	0.96	0.70	0.92	0.90
7	case	0.48	0.93	0.56	0.94	0.94	0.93	0.58	0.73	0.51
	control	0.50	0.96	0.79	0.96	0.94	0.98	0.63	0.91	0.87
8	case	0.45	0.96	0.77	0.94	0.87	0.90	0.58	0.78	0.80
	control	0.47	0.97	0.84	0.94	0.91	0.94	0.70	0.91	0.93
9	case	0.48	0.95	0.57	0.77	0.68	0.69	0.57	0.58	0.42
	control	0.44	0.95	0.74	0.95	0.91	0.93	0.61	0.76	0.81
11	case	0.49	0.94	0.76	0.97	0.89	0.95	0.67	0.76	0.77
	control	0.46	0.92	0.69	0.93	0.88	0.90	0.63	0.68	0.67
12	case	0.51	0.95	0.74	0.97	0.85	0.93	0.67	0.79	0.74
	control	0.47	0.93	0.62	0.94	0.94	0.93	0.70	0.71	0.65
13	case	0.44	0.91	0.57	0.93	0.86	0.88	0.56	0.71	0.64
	control	0.50	0.96	0.84	0.98	0.95	0.97	0.64	0.92	0.88
14	case	0.45	0.94	0.80	0.95	0.92	0.94	0.74	0.81	0.71

	control	0.45	0.96	0.82	0.96	0.93	0.95	0.82	0.88	0.89
'array' pair means (SE)										
	case	0.47 (0.01)	0.94 (0.004)	0.64 (0.04)	0.89 (0.03)	0.83 (0.04)	0.85 (0.04)	0.57 (0.04)	0.71 (0.04)	0.63 (0.05)
	control	0.46 (0.01)	0.95 (0.004)	0.75 (0.03)	0.95 (0.005)	0.93 (0.01)	0.94 (0.01)	0.65 (0.03)	0.83 (0.03)	0.82 (0.03)
'sequenom' pairs										
15	case	0.44	0.96	-	-	-	-	-	-	N/A
	control	0.43	0.98	0.78	0.94	0.87	0.85	0.68	0.83	N/A
16	case	0.42	0.93	0.65	0.95	0.81	0.87	0.66	0.73	N/A
	control	0.43	-	0.93	0.99	0.94	-	0.60	-	N/A
17	case	0.43	0.96	0.71	0.96	0.86	0.88	-	0.83	N/A
	control	0.43	0.92	0.78	0.95	0.86	0.98	-	0.82	N/A
18	case	0.41	0.92	-	-	-	0.56	-	-	N/A
	control	0.45	0.95	-	-	-	-	-	-	N/A
19	case	0.44	0.93	-	0.9	0.8	0.79	0.57	0.73	N/A
	control	0.44	0.97	0.83	0.95	0.91	0.93	0.79	0.90	N/A
20	case	0.43	0.96	-	0.75	0.59	0.53	0.38	0.64	N/A
	control	0.44	0.98	0.87	0.96	0.94	0.98	0.77	0.92	N/A
21	case	0.47	0.96	0.74	0.95	0.93	0.95	0.79	0.86	N/A
	control	0.46	0.95	0.77	0.95	0.86	0.93	0.71	0.77	N/A
22	case	0.43	0.94	0.66	0.93	0.80	0.92	0.54	0.72	N/A
	control	0.43	0.96	0.76	0.97	0.93	0.95	0.81	0.86	N/A
23	case	0.42	0.92	0.36	0.74	0.61	0.61	0.38	0.44	N/A
	control	0.44	0.96	0.73	0.95	0.87	0.88	0.66	0.78	N/A
24	case	0.46	0.96	0.88	0.97	0.93	0.94	0.86	0.91	N/A
	control	0.45	0.96	0.75	0.98	1.0	0.99	0.45	0.92	N/A
25	case	0.42	0.98	0.55	0.73	0.69	0.61	0.57	0.54	N/A
	control	0.43	0.99	0.92	0.97	0.96	0.99	-	-	N/A
26	case	0.45	0.97	0.84	0.97	0.91	0.97	0.87	-	N/A
	control	0.41	0.94	0.78	0.82	0.75	0.86	-	-	N/A
'sequenom' pair means (SE)										
	case	0.43 (0.01)	0.95 (0.01)	0.64 (0.07)	0.88 (0.05)	0.79 (0.05)	0.82 (0.07)	0.63 (0.07)	0.70 (0.07)	N/A
	control	0.44 (0.004)	0.96 (0.004)	0.78 (0.02)	0.96 (0.004)	0.91 (0.02)	0.93 (0.02)	0.70 (0.05)	0.85 (0.02)	N/A

Supplementary Table 4: Beta values for *IL32* Sequenom EpiTYPER assay units, in comparison to beta values from the significantly differentially methylated *IL32* HM27 array probe. The array probe was captured by the Sequenom assay unit labeled CpG_10. N/A = not assayed. "-" = missing data (did not pass quality control).

		Sequenom assay unit									HM27 array probe
Pair #	sample type	CpG_1	CpG_2	CpG_3	CpG_4	CpG_5	CpG_6.7	CpG_8	CpG_9	CpG_10	cg18350391
'array' pairs											
2	case	0.94	0.89	0.69	0.87	0.67	0.86	0.85	0.20	0.12	0.31
	control	0.85	0.88	0.49	0.87	0.63	0.77	0.78	0.27	0.13	0.48
3	case	0.86	0.90	0.96	0.84	0.47	0.70	0.71	0.04	0.03	0.07
	control	-	-	-	-	-	-	-	-	-	0.37
5	case	0.95	0.91	0.96	0.84	0.72	0.87	0.85	0.39	0.22	0.41
	control	0.95	0.89	0.95	0.89	0.83	0.83	0.87	0.55	0.37	0.54
6	case	0.88	0.89	0.5	0.85	0.65	0.80	0.80	0.23	0.09	0.29
	control	0.93	0.90	0.96	0.87	0.81	0.89	0.88	0.55	0.31	0.33
7	case	0.90	0.87	0.66	0.83	0.60	0.82	0.77	0.18	0.04	0.24
	control	0.87	0.87	0.94	0.78	0.60	0.81	0.79	0.39	0.19	0.33
8	case	0.91	0.89	0.66	0.82	0.72	0.83	0.79	0.43	0.25	0.49
	control	0.87	0.89	0.97	0.86	0.74	0.92	0.91	0.59	0.57	0.47
9	case	0.86	0.85	0.68	0.76	0.59	0.79	0.76	0.28	0.18	0.30
	control	0.91	0.93	0.65	0.85	0.62	0.82	0.76	-	0.18	0.47
11	case	0.81	0.88	0.67	0.79	0.47	0.74	0.69	0.13	0.10	0.22
	control	0.69	0.82	0.63	0.80	0.42	0.74	0.63	0.12	0.06	0.14
12	case	0.85	0.83	0.53	0.78	0.46	0.87	0.88	0.27	0.08	0.37
	control	0.71	0.81	0.49	0.72	0.35	0.63	0.60	0.07	0.03	0.19
14	case	0.91	0.90	0.54	0.87	0.69	0.81	0.83	0.17	0.07	0.24
	control	0.80	0.81	0.90	0.73	0.50	0.72	0.69	0.43	0.27	0.44
'array' pair means (SE)											
	case	0.88 (0.02)	0.88 (0.01)	0.68 (0.05)	0.82 (0.01)	0.60 (0.03)	0.81 (0.02)	0.79 (0.02)	0.23 (0.04)	0.12 (0.02)	0.29 (0.04)
	control	0.83 (0.03)	0.86 (0.01)	0.79 (0.08)	0.81 (0.02)	0.61 (0.06)	0.79 (0.03)	0.77 (0.04)	0.37 (0.07)	0.24 (0.06)	0.37 (0.05)

‘sequenom’ pairs											
15	case	0.92	0.9	0.96	0.88	0.69	0.86	0.78	0.29	0.11	N/A
	control	0.98	0.9	0.19	0.83	0.79	0.90	0.88	0.40	0.14	N/A
16	case	0.94	0.86	0.67	0.82	0.64	0.83	0.80	0.13	0.05	N/A
	control	0.89	0.90	0.73	0.87	0.63	0.87	0.84	-	0.02	N/A
17	case	0.82	0.82	0.68	0.77	0.52	0.77	0.74	0.20	0.08	N/A
	control	1	0.92	0.99	-	-	0.97	0.81	0.21	0	N/A
18	case	-	-	-	-	-	-	-	-	-	N/A
	control	0.90	0.88	0.49	0.86	0.78	0.87	0.84	0.21	0.06	N/A
20	case	0.83	0.94	0.33	-	0.35	0.72	0.59	0.07	0	N/A
	control	0.90	0.84	0.67	0.84	0.69	0.77	-	0.37	0.16	N/A
21	case	0.77	0.83	0.37	0.79	0.40	0.67	0.70	0.12	0.02	N/A
	control	0.82	0.83	0.91	0.73	0.48	0.72	0.67	0.22	0.12	N/A
22	case	0.94	0.89	0.98	0.84	0.81	0.94	0.92	0.51	0.34	N/A
	control	0.86	0.91	0.53	0.84	0.67	0.86	0.80	0.19	0.07	N/A
23	case	0.81	0.84	0.67	0.83	-	0.70	0.67	0.05	0.04	N/A
	control	0.80	0.82	0.69	0.72	0.51	0.71	0.71	0.16	0.08	N/A
24	case	0.87	0.87	0.33	0.80	0.64	0.82	0.74	0.35	0.2	N/A
	control	0.81	0.91	0.48	0.61	-	0.73	0.65	0.43	0.12	N/A
25	case	0.81	0.89	0.98	0.82	0.46	0.73	0.74	0.03	0.03	N/A
	control	0.99	0.98	0.99	0.88	0.74	0.92	0.92	0.62	0.36	N/A
26	case	-	-	-	-	-	-	-	0.52	0.19	N/A
	control	0.87	-	0.66	0.76	0.68	0.87	0.75	0.14	0.02	N/A
‘sequenom’ pair means (SE)											
	case	0.87 (0.03)	0.87 (0.01)	0.71 (0.11)	0.81 (0.01)	0.59 (0.05)	0.80 (0.03)	0.77 (0.03)	0.23 (0.06)	0.12 (0.04)	N/A
	control	0.89 (0.03)	0.89 (0.02)	0.63 (0.12)	0.81 (0.03)	0.66 (0.06)	0.83 (0.04)	0.80 (0.04)	0.30 (0.07)	0.14 (0.05)	N/A
